# Study on differentially expressed genes between stage M and stage MS neuroblastoma

**DOI:** 10.3389/fonc.2022.1083570

**Published:** 2023-01-13

**Authors:** Yuying Wu, Jun Zhang

**Affiliations:** Department of Surgical Oncology Children’s Hospital of Chongqing Medical University, National Clinical Research Center for Child Health and Disorders, Ministry of Education Key Laboratory of Child Development and Disorders, Chongqing Key Laboratory of Pediatrics, Chongqing, China

**Keywords:** neuroblastoma, stage MS, DEGs, immunohistochemistry, apoptosis, ferroptosis

## Abstract

**Objective:**

To search for the DEGs between stage MS NB and stage M NB and speculate the possible mechanism of spontaneous regression of stage MS NB.

**Materials and methods:**

The NB datasets GSE49710 and GSE45547 in the GEO database were selected to screen the DEGs between children with NB stage MS vs. stage M, < 18 months. GO enrichment and KEGG pathway analysis of DEGs was performed using DAVID. The intersecting genes among DEGs and RCD-related genes were selected, and their survival roles and functions were assessed. We then used the collected clinical samples to validate the expression of these genes at the protein level using IHC methods and further analysis to explore their role.

**Results:**

BIRC5, SLCO4A1, POPDC3, and HK2 were found to be downregulated in stage MS NB and related to apoptosis. BIRC5 and HK2 also participate in autophagy. The TF gene is upregulated in stage MS NB and related to ferroptosis. The above five genes are closely related to the survival of children with NB. And the expression levels of all five genes at the protein level were verified by IHC to be consistent with the results of the preliminary screening described above.

**Conclusion:**

BIRC5, SLCO4A1, POPDC3, HK2 and TF are expected to become new important indicators to predict the prognosis of NB and can be used as the basis for further explored the benign prognosis and spontaneous regression mechanism of stage MS NB.

## Introduction

Neuroblastoma (NB) is the most common extracranial solid tumor in children. It most often occurs in the adrenal gland, accounting for approximately 15% of all pediatric cancer-related deaths (1, 2). Although active multimodal treatment is adopted, the prognosis of children in different stages of disease still varies greatly (3). At present, it is generally believed that children with distant metastasis often have a poor prognosis. Nevertheless, there is a special stage–stage MS–that exhibits a phenomenon of spontaneous regression and a good prognosis (4).

To date, it is believed that the spontaneous regression of stage MS NB may involve the following mechanisms: (1) neurotrophin deficiency, (2) telomerase inactivation, (3) humoral or cellular immunity and (4) changes in epigenetic regulation(4). However, these mechanisms are still in the preliminary research stage, and the spontaneous regression of stage MS NB has not been fully explained.

The modes of cell death include accidental cell death and regulatory cell death (RCD). At present, many evidences show that RCD is the main factor of cell death, which is a spontaneous mode of cell death, including apoptosis, autophagy, ferroptosis and other modes, and is closely related to tumor progression (5, 6). Here, we speculate that the spontaneous regression of stage MS NB may be related to RCD.

## Materials and methods

### Search for RCD-related genes

The keywords “apoptosis”, “autophagy”, “ferroptosis”, “pyroptosis”, and “necroptosis” were entered into the Molecular Signatures Database (MSigDB) to search for RCD-related genes. We searched for additional autophagy-related genes in a dedicated Human Autophagy Database (HADb) and found additional ferroptosis-related genes on the FerrDb website (http://www.zhounan.org/ferrdb/), which is a database with information on regulators and markers of ferroptosis and ferroptosis-disease associations (7).

### Identification of shared differentially expressed genes (DEGs)

Two NB datasets GSE49710 and GSE45547 were selected from the Gene Expression Omnibus (GEO) database. There were 498 NB samples in the GSE49710 dataset, which was submitted by Wang C et al. (8). There were 649 NB samples in GSE45547, which was submitted by Kocak H et al. (9). The two datasets are based on the platform GPL16876 Agilent-020382 Human Custom Microarray 44k (Feature Number version). We only included children with stage MS and stage M (<18 months) for DEG analysis to exclude age interference. The detailed dataset information of the two NB datasets is shown in **Table 1**.

**Table 1:** Details for datasets from GEO.

GEO	Platform	Sample	Stage M (<18months)	Stage MS	Submission	Update	Author
GSE49710	GPL16876	NB	52	53	Aug9,2013	Oct15,2015	Zhang H
GSE45547	GPL16876	NB	66	78	Mar27,2013	Apr16,2013	Kocak H

Using the limma package in R software (version 4.1.2), DEGs between stage MS and stage M (<18 months) were screened in two datasets, and the parameters for judging the difference were set as adj. P < 0.05 and | log2FC | > 1. The difference between upregulation and downregulation refers to stage MS relative to stage M. The network analysis tool Venny (https://bioinfogp.cnb.csic.es/tools/venny/index.html) was chosen, and the intersecting DEGs were obtained *via* a Venn diagram.

### Functional analysis of common DEGs

The Database for Annotation, Visualization and Integrated Discovery (DAVID) online tool was used to conduct Gene Ontology (GO) enrichment analysis and Kyoto Encyclopedia of Genes and Genome (KEGG) pathway analysis on the screened common DEGs. The GO analysis included biological process (BP), cellular component (CC), and molecular function (MF) categories. Then, the enrichment results are visualized.

### Survival analysis

The R2 (https://hgserver1.amc.nl/cgi-bin/r2/main.cgi) online website was used to analyze the effects of age variables and intersecting DEGs on the survival rate of children, determine the DEGs that are closely related to survival, and draw Kaplan−Meier (K-M) survival curves.

### Clinical materials and immunohistochemistry (IHC)

To determine DEGs expression in NB, we performed IHC staining of DEGs in the NB tissues of 21 children with M stage and 9 children with MS stage. The study, authorized by the ethics committee of Children’s Hospital of Chongqing Medical University. NB tumor tissues were embedded in paraffin and severed into slices (4 mm). After dewaxing, hydration and antigen repair, each sample was titrated with five primary antibodies: Anti-BIRC5 (No.380719, ZENBIO, China), Anti-SLCO4A1 (No. YT3221, Immunoway), Anti-POPDC3 (No. 11800-1-AP, Proteintech), Anti-HK2 (No. R24552, ZENBIO, China) and Anti-TF (No. R25969, ZENBIO, China), respectively. Then incubated overnight at 4° C. Then the steps of incubation with the secondary antibody Goat anti-Rabbit IgG (No. PV-9001, ZSGB-BIO, China), DAB (No. ZLI-9018, ZSGB-BIO, China) staining and blocking were performed, and the staining effect was observed under the microscope. The mean Integrated Optical Density (IOD) value of each slice was determined using Image-Pro Plus 6.0 software. Three to five fields of view were taken for each slice and the average IOD value was taken as the final IOD value for that slice. Subsequently, the IOD values between the M and MS groups for each protein were tested for differences using Student’s t test.

## Results

### The genes involved in RCD were selected

A total of 430 apoptosis gene sets which contained 790 apoptosis-related genes were retrieved from the MSigDB website, and one of them, M12113 (10), was selected. For autophagy-related genes, we selected the genes in all 20 gene sets retrieved from MSigDB, retrieved an additional 232 genes in the HADB database, deleted the duplicate genes, and finally identified 727 autophagy-related genes. A gene set which contains 40 ferroptosis-related genes (M39768) was retrieved from MSigDB. In addition, ferroptosis-related genes in the FerrDb website were downloaded, duplicate genes were deleted, and 275 ferroptosis-related genes were finally screened. MSigDB has only one pyroptosis gene set, M41804, which contains 27 pyroptosis genes. Finally, we retrieved the only necroptosis gene set, M24779, which contains 8 necroptosis genes.

### Identification of the common DEGs

**Figure 1:**
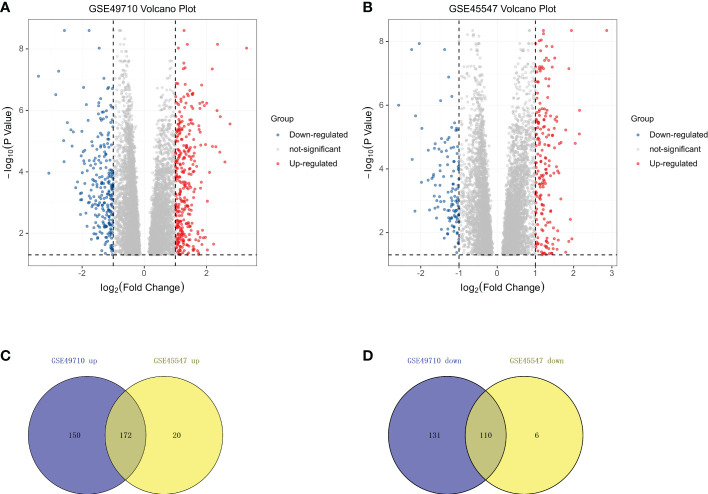
DEGs in two datasets from the GEO. **(A)** Volcano plots showing the DEGs in GSE49710. **(B)** Volcano plots showing the DEGs in GSE45547.The red dots represent upregulated genes, blue dots represent downregulated genes, and gray dots indicate genes with no significant differences. **(C)** Venn diagram showing the intersection of upregulated genes. **(D)** Venn diagram showing the intersection of downregulated genes. All DEGs are screened based on an Adjust P value < 0.05 and |Fold Change| > 1.

The NB expression microarray datasets GSE49710 and GSE45547 were background corrected and normalized, and the DEGs between stage MS and stage M (<18 months) samples in the two datasets were screened by using the limma package in R software. There were 563 DEGs, including 322 upregulated and 241 downregulated DEGs, in GSE49710. In GSE45547, there were 308 DEGs, of which 192 were upregulated and 116 were downregulated **(** **Table 2** **)**. A volcano plot was made for these DEGs of the two datasets **(** **Figures 1A, B** **)**. Then, the Venny website was used to intersect the upregulated DEGs and downregulated DEGs separately, and 172 common upregulated DEGs and 110 common downregulated DEGs between the two datasets were obtained **(** **Figures 1C, D** **)**.

**Table 2:** DEGs screened from two datasets.

	Up-regulated	Down-regulated	Sum
GSE49710	322	241	563
GSE45547	192	116	308
Common genes	172	110	282

### GO enrichment and KEGG pathway analysis of DEGs

**Figure 2:**
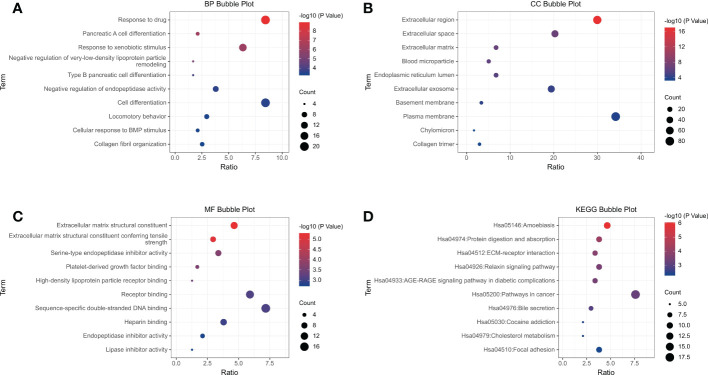
GO function and KEGG pathway analysis of 282 common DEGs. Analysis of **(A)** Biological Process, **(B)** Cellular Component, and **(C)** Molecular Function. **(D)** KEGG analysis showed the enriched pathways. Each functional section shows 10 terms.

To further understand the functions and pathways of the intersecting DEGs, we conducted GO enrichment and KEGG pathway analyses and visualized the results. In terms of BPs, DEGs were significantly enriched in the response to drug, pancreatic A cell differentiation, the response to xenobiotic stimulus and other processes **(** **Figure 2A** **)**. In the CC category, DEGs were mainly involved in extracellular region, extracellular space, blood microparticle, endoplasmic reticulum lumen, etc. **(** **Figure 2B** **)**. The analysis of MF showed that DEGs were mainly enriched in extracellular matrix structural constituents, extracellular matrix structural constituents conferring tensile strength, serine-type endopeptidase inhibitor activity, platelet-derived growth factor binding, etc. **(** **Figure 2C** **)**. In addition, KEGG pathway analysis showed that DEGs were mainly involved in amebiasis, protein digestion and absorption, ECM-receptor interaction, the relaxin signaling pathway, and the AGE-RAGE signaling pathway in diabetes complications **(** **Figure 2D** **)**.

### Survival analysis based on the intersecting genes

**Figure 3:**
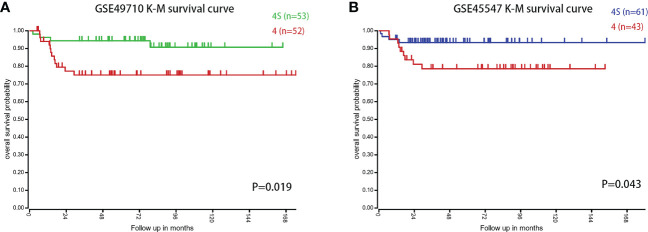
The survival difference between children with stage MS and stage M NB (<18 months) in the two datasets. **(A)** K-M survival curves of children with stage MS and stage M (<18 months) NB in GSE49710. **(B)** K-M survival curves of children with stage MS and stage M (<18 months) NB in GSE45547.

**Figure 4:**
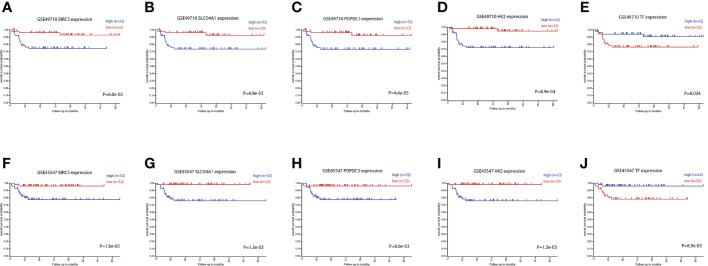
Survival curves of patients grouped by BIRC5, SLCO4A1, POPDC3, HK2 and TF expression in the two datasets. **(A–E)** The prognostic value of BIRC5, SLCO4A1, POPDC3, HK2 and TF in the GSE49710 dataset. **(F–J)** The prognostic value of BIRC5, SLCO4A1, POPDC3, HK2 and TF in the GSE45547 dataset. Gene expression levels are grouped by median.

**Figure 5:**
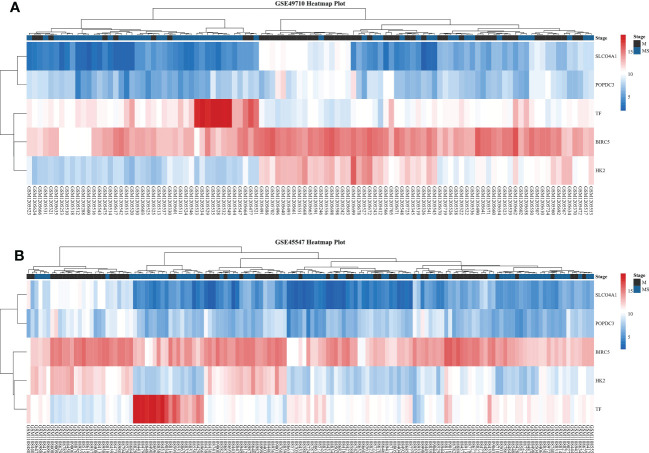
Heatmaps of five DEGs in two datasets. **(A)** Heatmap of five DEGs in GSE49710. **(B)** Heatmap of five DEGs in GSE45547. From red to blue, the expression level of the DEGs in the sample gradually decreases.

We first compared the survival differences between children with stage MS NB and stage M (<18 months) NB in the two datasets. The results confirmed that there were definite survival differences between the two groups, which provided a basis for the selection and analysis of DEGs (**Figures 3A, B**). Then, we took the intersection of all DEGs and RCD genes **(** **Table 3** for the results) to analyze the effects of these intersecting genes on survival. The results showed that the expression levels (grouped by median) of five genes (BIRC5, SLCO4A1, POPDC3, HK2, and TF) were closely related to the survival of children in both datasets (the K-M survival curves are shown in **Figures 4A–J** **)**. The heatmap of these five genes is shown in **Figures 5A, B**. Low expression levels of BIRC5, SLCO4A1, POPDC3 and HK2 are favorable factors in terms of the prognosis of children, while high expression of TF is a favorable factor. In addition, GO enrichment and KEGG pathway analysis of the above five genes showed that both TF and HK2 participate in the HIF-1 signaling pathway.

**Table 3:** Intersection genes of DEGs and RCD genes.

RCD	Count	Intersection with up-regulated DEG	Intersection with down-regulated DEG
apoptosis	790	AKAP7,MYLK,KANK1,MGST1	BIRC5,CDCA5,HK2,POPDC3,SLC18A3,SLCO4A1
autophagy	727	EXOC4,EPAS1,COL3A1,FN1,COL1A1	BIRC5,HK2,DYNC1I1
ferroptosis	275	TF,EPAS1,MAP3K5	/
pyroptosis	27	/	/
necroptosis	8	/	/

### Detection of antibodies using IHC

**Figure 6:**
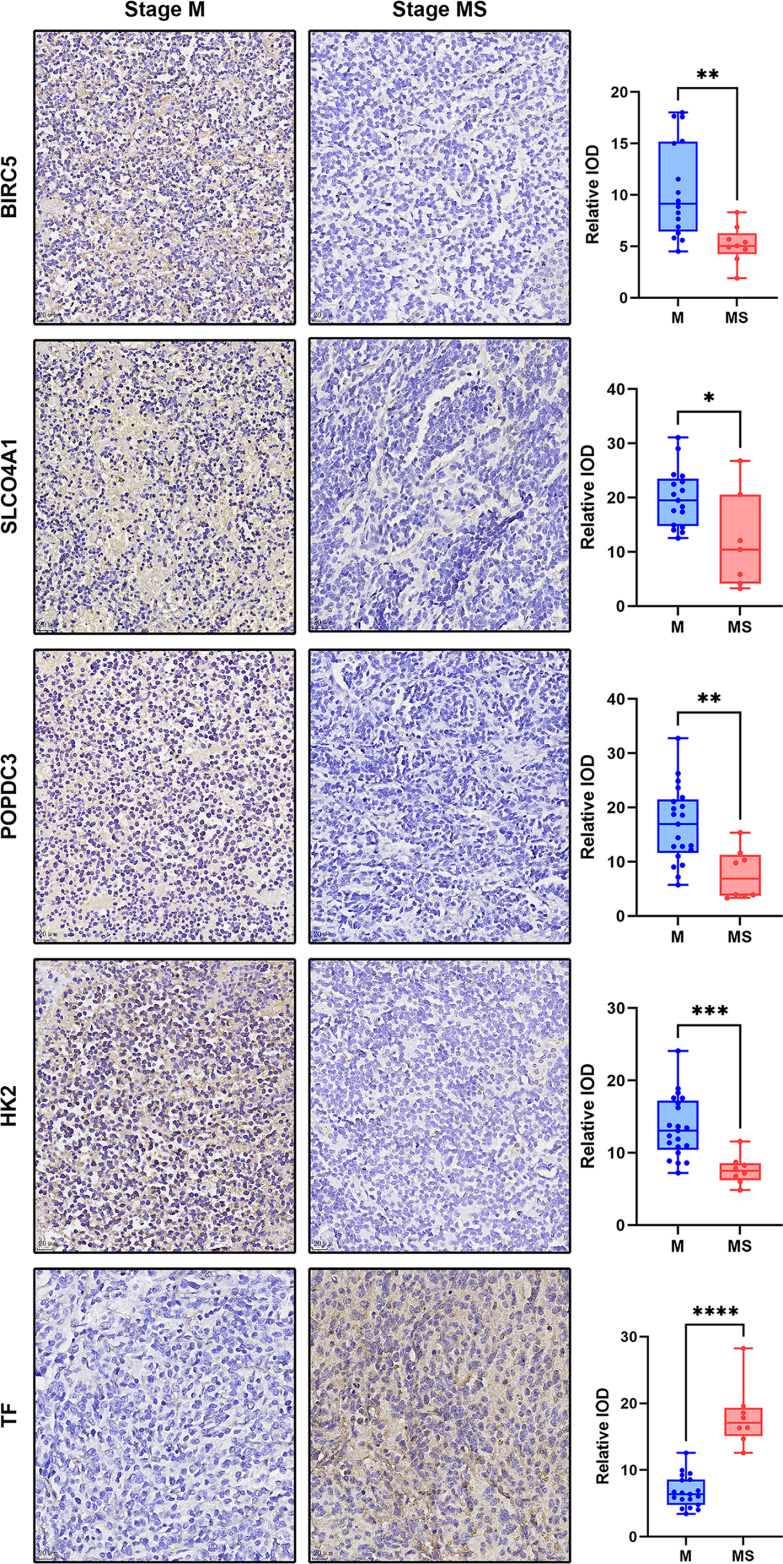
Display of IHC dyeing effect of DEGs and the dot plot of relative IOD values of each group. The magnification of the IHC images was 40×, scale bar=20μm. The dot plot shows the difference of IOD values between samples of each protein in M and MS stage. *p < 0.05, **p < 0.01, ***p < 0.001, ****p < 0.0001.

In total, we collected a sample of 30 eligible children with NB, including 21 with stage M and 9 with stage MS. All children were ≤18 months of age and had intact preserved paraffin tissue sections. Slicing thickness of 4μm. Quantitative analysis of IHC showed that the expression of five genes, BIRC5, SLCO4A1, POPDC3, HK2 and TF, were significantly different in M- and MS-stage NB samples**(** *P*<0.05, **Figure 6** **)**. Among them, BIRC5, SLCO4A1, POPDC3 and HK2 were significantly higher in the M-stage samples than in the MS-stage, while TF was significantly higher in the MS-stage samples, which is consistent with the results of the preliminary screening described above.

## Discussion

Apoptosis is the most deeply studied RCD mode at present. The process of apoptosis eventually activates caspase protein, resulting in cell death (11). The weakening of apoptosis often leads to tumorigenesis, and the overexpression of antiapoptotic oncogenes such as BCL-2/BCL-XL, MCL1 or the IAP proteins is conducive to the survival of tumor cells (12). The results of our study showed that BIRC5, SLCO4A1, POPDC3, and HK2 were involved in the process of apoptosis.

BIRC5, also known as survivin, is the strongest inhibitor of apoptosis found thus far (13), and its main role is related to the inhibition of caspase activity (14). The expression of BIRC5 is downregulated during normal tissue development and cannot be detected in most final differentiated adult tissues (15). In a variety of tumors, such as ovarian cancer, breast cancer, colorectal cancer (16) and renal cell carcinoma (17), the expression is increased and is positively correlated with metastasis and a low survival rate. SLCO4A1 is proved to be highly expressed in colorectal cancer and affect prognosis (18), but its role in NB has not been studied. POPDC3 belongs to the Popeye domain containing (POPDC) family, and is a recently discovered cyclic 3′,5′-adenosine monophosphate (cAMP) effector protein (19). Recent studies have shown that the expression level of POPDC3 is closely related to the cell proliferation, metastasis and prognosis of a variety of cancers, especially gastric cancer, and can be used as a potential cancer treatment target (20–22). In addition, research on POPDC3 covers topics including muscular dystrophy, cardiac function and other aspects (23, 24); research on POPDC3 in NB is lacking, but this is a topic worthy of in-depth exploration. HK2, hexokinase 2, is an important glycolytic enzyme that catalyzes the conversion of glucose to glucose 6-phosphate. Studies have shown that the expression level and activity of HK2 in metastatic NB tumor tissues are higher than those in local NB tumor tissues, suggesting that HK2 plays an important role in the formation of the malignant phenotype of NB and affects the progression of the disease (25).

This study showed that BIRC5, SLCO4A1, POPDC3, and HK2 were significantly downregulated in stage MS NB and affected the survival rate of children, indicating that the low expression of these four genes is a factor conducive to tumor regression. All these factors are involved in the process of apoptosis. We propose that the spontaneous regression of stage MS NB is closely related to the process of apoptosis, and the above four genes play an important role in this process of apoptosis.

In addition, our results show that BIRC5 and HK2 are also involved in autophagy. Autophagy is a cellular pathway involved in the degradation of proteins and organelles, and it is the mechanism of cell survival under stress stimulation. Currently, autophagy is considered to play a dual role in cancer. It inhibits the growth of benign tumors but promotes the growth of advanced tumors. Many research groups have established autophagy as a potential therapeutic target for cancer (26). A study showed that some autophagy-related genes were differentially expressed between stage MS and stage M (<18 months) NB. The results showed that autophagy inhibited the progression and promoted the spontaneous regression of NB (27).

It is generally believed that apoptosis and autophagy are not completely isolated processes (28). BIRC5 is an important molecule connecting the two processes (29). BIRC5 is positively regulated by the AKT/mTOR pathway to inhibit autophagy and apoptosis and promote tumor cell survival (14). The degradation of BIRC5 releases bound beclin-1, enhances autophagy and induces cell death, which also shows that the increase in BIRC5 levels is closely related to the inhibition of autophagy (30). In addition, some studies have pointed out that HK2 plays the same role as BIRC5 and participates in the process of autophagy and apoptosis through the AKT/mTOR pathway. Targeting HK2 to treat cancer is also a promising strategy (31–33). This is consistent with our results (BIRC5 and HK2 are downregulated genes), so we speculate that the spontaneous regression of stage MS NB is likely to be related to the autophagy and apoptosis processes related to BIRC5 and HK2 because when the expression levels of BIRC5 and HK2 are low, the inhibition of apoptosis and autophagy by other factors will be relieved.

TF, transferrin, was the only gene upregulated in stage MS NB in our results. It is a key molecule involved in ferroptosis. Its function is to transport iron from the site where heme is absorbed and degraded to the site where heme is stored and utilized. TF and its receptor TFR can regulate the process of ferroptosis directly or indirectly (34).

Ferroptosis is a newly defined RCD mode that was first proposed by Scott J. Dixon and others in 2012 (35). Ferroptosis is associated with a variety of pathological conditions, such as acute tissue injury, infection, inflammation, cancer and neurodegeneration (36, 37). The molecules involved in ferroptosis include Nrf2, p53, heme oxygenase‐1, FANCD2, and BECN1, and the role of ferroptosis in breast cancer, hepatocellular carcinoma, renal cancer and other diseases has been studied (38). Our study shows that the TF gene is differentially expressed between stage MS and stage M (<18 months) NB and affects the survival of children. We propose that the spontaneous regression of stage MS NB involves ferroptosis regulation and that TF plays an important role in this process.

GO enrichment and KEGG pathway analysis revealed that the DEGs were mainly involved in response to drug, response to xenobiotic stimulus and other processes, as well as in amebiasis, protein digestion and absorption, ECM-receptor interaction and other pathways. Among the above five DEGs that affect survival, both TF and HK2 are involved in the HIF-1 signaling pathway; TF is involved in the process of cell ferroptosis; and HK2 is involved in the processes of autophagy and apoptosis.

HIF-1 is a member of the hypoxia inducible factor protein family, which can help cells adapt to the hypoxic environment. HIF-1 is a signaling center, and its role in cancer has been fully described. Inhibiting HIF-1 is a promising treatment strategy for cancer and cancer-related inflammation (39, 40). The function of HIF-1 in NB has also been widely studied. The increased expression and activity of HIF-1 promotes the proliferation, invasion and migration of NB cells, which is positively correlated with the malignant degree of NB (41). Some studies have shown that HIF-1/HK2 can synergistically promote the development of breast cancer (42). However, this synergistic effect has not been studied in NB. In addition, in studies of different kinds of cells, it has been shown that an increase in HIF-1 levels can inhibit ferroptosis, and inactivation of this pathway can induce ferroptosis (43, 44). In osteoclasts, elevated HIF-1 expression can inhibit not only ferroptosis but also autophagy (45). Therefore, we think that the spontaneous regression of stage MS NB involves autophagy and ferroptosis regulated by TF, HK2 and the HIF-1 signaling pathway.

### Summary

Our study showed that there were significant differences in the expression levels of BIRC5, SLCO4A1, POPDC3, HK2 and TF between stage MS and stage M (<18 months) NB. Survival analysis showed that they were closely related to the prognosis of children with NB. GO enrichment and KEGG pathway analyses of DEGs further revealed their functions and pathways. These genes may become potential markers for improving NB diagnosis, optimizing treatment and predicting prognosis. Since these genes are important genes involved in the RCD mode, they also provide a breakthrough point for further exploration of the spontaneous regression of stage MS NB. Moreover, there are few studies on the role of these genes in NB. It is necessary for us to further verify and explore the potential functions and pathways of these genes.

## Data availability statement

The datasets presented in this study can be found in online repositories. The names of the repository/repositories and accession number(s) can be found in the article/supplementary material.

## Ethics statement

The studies involving human participants were reviewed and approved by Children’s Hospital of Chongqing Medical University. Written informed consent to participate in this study was provided by the participants’ legal guardian/next of kin. Written informed consent was obtained from the minor(s)’ legal guardian/next of kin for the publication of any potentially identifiable images or data included in this article.

## Author contributions

All authors listed have made a substantial, direct, and intellectual contribution to the work, and approved it for publication.
